# Report of a Case of Typhoid Fever, with Remarks

**Published:** 1867-10

**Authors:** Charles Pinckney

**Affiliations:** Atlanta, Ga.


ATLANTA

### Medical and Surgical Journal.

NEW SERIES.

VOL. VIII. OCTOBER, 1867. No. 8.

### ORIGINAL COMMUNICATIONS.

### ARTICLE I.

*Report of a Case of Typhoid Fever, with Remarks.* By CHARLES PINCKNEY, M. D., of Atlanta, Ga.

An interesting case of Typhoid Fever came under my notice in the year 1859, while engaged in the practice of medicine in the rice-growing region of South Carolina. As it presented certain conditions and peculiarities which I have never observed before or since, a brief sketch of its history, prepared from notes taken at the time, may not prove uninteresting to the profession. The patient, Dolly, aged eight years, the property of Mrs. E. P. Pinckney, Ashepoo, S. C., was seen by me, for the first time, on the 7th of June 1859. The earlier symptoms were so nearly analogous to Billious Remittent, that I directed the usual treatment for that disease. It was not long, howevor, before sufficient evidence was presented to prove the incorrectness of my diagnosis. The case was one of Typhoid Fever. This, I confess, is not the only instance where I have made a similar mistake; for to those who have practiced amid the malarial swamps of lower Carolina, the task would frequently be a difficult one to determine the precise nature and grade of fevers in their incipiency. The simple Continued, would seem, in certain cases, to degenerate into Typhoid, and a fever which had observed all the rules of periodicity for an indefinite number of days, has been known to assume the enteric form, wherein was developed all the symptoms peculiar to that disease, terminating in perforation of the intestines, as revealed by post-mortem examination. Whether the latter was merely engrafted upon the former, or whether it was indeed a degeneration thereof is a question not yet satisfactorily settled. But to return to the case under consideration.

On the 6th of July the patient was attacked suddenly with the most excruciating abdominal pains, located apparently in the region of the cœcum. She seemed to be laboring under acute peritonitis. Calomel and Opium, in small doses, were now resorted to, and a blister applied over the seat of pain. These means failing to arrest inflammation, ice was next freely used internally, and externally over the abdomen. Diarhœa soon set in, of the most debilitating character—the evacuations being frequent, watery, and very offensive. Pulse became small, thready, and so frequent that it could barely be counted. Brandy produced no perceptible reaction—though administered in considerable quantity and at short intervals. The patient continued growing worse, and lapsed into a partially comatose condition. At this stage I ordered:

Spts. Terebinth, gtt. xxx.
Muc. g. Acaciæ, ℥ ss.
M.

S—To be repeated every hour.

After the fourth dose her condition improved somewhat; and, attributing it to the effect of the Terpentine, that medicine, in smaller quantity, combined with other remedies, was continued until the evacuations became less frequent and more natural. Fever declined in proportion to the abatement of the abdominal symptoms. On the third day but little pain was felt upon pressure over the right Illiac region; her appetite was coming back; she was evidently better.

At this juncture the patient passed, during an evacuation, a large portion of what appeared to be the mucous membrane of the intestine, considerably thickened by previous inflammation. A part of the same substance which had escaped per anum, but remained attached by one extremity to the external terminus of the rectum, was clipped off and preserved as something curious. Its consistency was firm. It was six inches in length, and a perfect cylinder. Without the closest examination it might readily have ben mistaken, even by professional men, for a section of intestine. My friend, Dr. G. M Rivers, of Walterboro, S. C., may have it still in his possession. Other smaller and entirely detached portions of the same material subsequently escaped, leaving the anus exceedingly sore and sensitive. Diarhœa again threatened to destroy the patient. Injections could not be retained for a moment; but the use of supporitories, retained in position by means of a T bandage, finally arrested the discharges. Irritation subsided and the evacuations became natural. Tonics and stimulants, in combination with diuretics, were now given—these last in order to remove an effusion of water which had occurred to some extent. Diet was particularly watched, and animal food administered as regularly as medicinal remedies. On the second of August she stood up and talked with me for a minute or two. I had no doubt she would recover. At daylight next morning the nurse informed me that "Dolly is dead."

The autopsy revealed some interesting as well as surprising facts. Upon opening the abdomen about two quarts of water escaped—being the effusion before alluded to. I then proceeded to examine the rectum, where disease was thought to have produced the principal lesion. Here the traces of previous inflammation and destruction were plain. A surface of three inches in length, by one and a-half inches in breadth of the anterior wall of that viscus had entirely sloughed away; and what is most remarkable, the free edges had become adherent to that portion of the bladder nearest the rectum. The external coat of the bladder had thus become, for the space of several inches, a part of the walls of the rectum—resembling mucous membrane, and was actually, in a mechanical view, performing the functions of that organ. This adhesion was so firm as to require some exertion to break it down. There was also direct communication, between the cœcum and sigmoid fixture, at a point where perforation and subsequent adhesion had occurred. The evidence of recent perforation could easily be observed at various points along the colon. The alimentary canal was examined throughout its entire length and found to be empty.

To what cause are we to assign the death of this patient? Surely not to perforation of the intestines; for nature had here so miraculously exerted here recuperative powers, that by healing here and by *substitutiny* there, she had completely remedied loss of structure. There was by no means a sufficient amount of fluid in the abdominal cavity to cause death by suffocation. My opinion is that death resulted from *inanition.* There was neglect, evidently, on the part of the nurse, in administering food in such quantity and at such times as had been directed—which is supported by the fact that the bowels were found empty.

The treatment in this case has not been given in detail. It was mainly expectant, and from the beginning the diet was so administered as to embrace as much nutriment as possible in the smallest compass—consisting of such substances as would more readily be absorbed by the stomach, thereby avoiding all offensive accumulations in the intestines. Had not this course been strictly followed it is evident that the patient would have died from extravasation of the peritoneum.

In reviewing the history of this case the mind is necessarily struck with the herculean efforts put forth by nature in the exercise of her recuperative powers. It should teach us never to despair. In the treatment of Typhoid Fever, with me "Hope springs eternal;" for though a patient has been brought by it to the verge of death he *may* yet recover —even after extensive intestinal perforations, or the absolute destruction of full one-third of the rectum.

N. B.—This post-mortem examination was witnessed by Drs. G. W. Rivers and F. W. Frazer, of Walterboro', S. C.

